# miRMaid: a unified programming interface for microRNA data resources

**DOI:** 10.1186/1471-2105-11-29

**Published:** 2010-01-14

**Authors:** Anders Jacobsen, Anders Krogh, Sakari Kauppinen, Morten Lindow

**Affiliations:** 1 The Bioinformatics Centre, Department of biology, University of Copenhagen, 2200 Copenhagen N, Denmark; 2 The Biotech Research and Innovation Centre BRIC), Department of biology, University of Copenhagen, 2200 Copenhagen N, Denmark; 3 Santaris Pharma A/S, Kogle Allé 6, Hørsholm, Denmark; 4 Copenhagen Institute of Technology, University of Aalborg, Ballerup, Denmark

## Abstract

**Background:**

MicroRNAs (miRNAs) are endogenous small RNAs that play a key role in post-transcriptional regulation of gene expression in animals and plants. The number of known miRNAs has increased rapidly over the years. The current release (version 14.0) of miRBase, the central online repository for miRNA annotation, comprises over 10.000 miRNA precursors from 115 different species. Furthermore, a large number of decentralized online resources are now available, each contributing with important miRNA annotation and information.

**Results:**

We have developed a software framework, designated here as miRMaid, with the goal of integrating miRNA data resources in a uniform web service interface that can be accessed and queried by researchers and, most importantly, by computers. miRMaid is built around data from miRBase and is designed to follow the official miRBase data releases. It exposes miRBase data as inter-connected web services. Third-party miRNA data resources can be modularly integrated as miRMaid plugins or they can loosely couple with miRMaid as individual entities in the World Wide Web. miRMaid is available as a public web service but is also easily installed as a local application. The software framework is freely available under the LGPL open source license for academic and commercial use.

**Conclusion:**

miRMaid is an intuitive and modular software platform designed to unify miRBase and independent miRNA data resources. It enables miRNA researchers to computationally address complex questions involving the multitude of miRNA data resources. Furthermore, miRMaid constitutes a basic framework for further programming in which microRNA-interested bioinformaticians can readily develop their own tools and data sources.

## Background

MicroRNAs (miRNAs) are short regulatory RNA molecules that are encoded in the genomes of animals, plants and viruses. They function as post-transcriptional regulators of mRNAs and have gained high interest due to their importance in many biological processes [1-3] and their potential as drug targets [4]. The relatively recent discovery and the main mechanism of action of miRNA-based regulation, which is based on Watson-Crick base pairing, has led to a recent explosion in algorithms, websites and databases that provide different data about microRNAs.

The large number of miRNAs discovered during the last couple of years has been supported by miRBase as the central clearing house for miRNA nomenclature and annotation [5,6]. At the miRBase web site, scientists can submit newly discovered miRNAs and information about sequences and homologies in other species. Today miRBase has become a central and highly useful website for scientists who search for information about specific miRNAs. A number of flat files in different formats are made available with each release of miRBase to support computational analysis. In addition to miRBase, a variety of miRNA data resources has been developed by other research groups. These include resources that deal with genomic contexts and evolutionary conservation of miRNAs (miROrtho [7], miRGen [8], miRfunc [9], microTranspoGene [10]), prediction and validation of miRNA targets (TargetScan [11], miRNAMap [12], microRNA.org [13], miRDB [14], miRecords [15], TarBase [16]) and biological functions and phenotypes of individual miRNAs (miR2Disease [17], DIANA-mirPath [18], MMIA [19]). These miRNA resources are primarily available online as point-and-click web sites.

It is currently a burdensome task to do an integrated computational analysis using data from one or more of the online miRNA resources. For each resource, it requires manually downloading raw data files (if available), understanding the sometimes arcane format and structure of the resource in question and finally, construction of a script to parse the content and various identifiers. The researcher has to go through all these steps, and repeat them each time a resource is updated. A more simple procedure would reduce errors, increase reproducibility of the scientific results and make the data analysis less labor-intensive. miRMaid is a software framework designed to eliminate the aforementioned preprocessing steps. It provides non-redundant, structured and inter-connected data that are accessible both through an object oriented interface (using the Ruby programming language) and as web-based resources that are accessible remotely using most computer programming languages. The web-based resources follow a set of design principles, Representational State Transfer (REST) [20], implying that every resource is uniquely and uniformly addressable using an URL. The effect is that the web resources can be accessed equivalently by computer programs and researchers using a web browser.

## Implementation

### Core architecture

**Figure 1:**
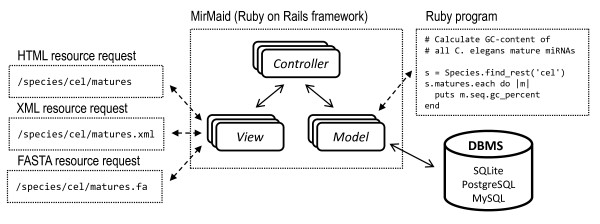
**Architecture overview**. miRMaid uses a Model-View-controller architecture. The model layer provides object oriented encapsulation of data stored in a relational database. The model layer can be efficiently and directly queried using the Ruby programming language. Each model is additionally exposed as a RESTful web resource. The data returned from a resource URL can be returned as HTML (suitable for web browsers), XML (suitable for computer programs) and for some resources also as FASTA sequence format.

miRMaid is built in the Ruby programming language using an open source web application framework, Ruby on Rails (RoR, http://www.rubyonrails.org). RoR allows rapid development of web applications in a Model-View-Controller (MVC) architecture, which isolates business logic from the user interface and facilitates program maintenance and scalability. In the RoR framework, data is stored in a relational database management system (SQLite, PostgreSQL and MySQL are currently supported in miRMaid) and encapsulated in an object-oriented model layer (Figure 1). The models are inter-connected and can be queried directly from the Ruby programming language. When miRMaid is deployed, it automatically (unless a specific miRBase version is stated) fetches the online raw data files from the current miRBase release. This data is restructured to yield the set of miRMaid core data models. An overview of these models and their associations is shown in Table 1.

**Table 1:** Models

Model	Description	Attributes	Relationships
Precursor	The miRNA precursor, processed from longer primary transcripts by endonucleases.	name, accession, description, sequence, comment	**Mature**,**GenomeContext, GenomePosition, Paper, PrecursorCluster**, Species, PrecursorFamily

Mature	The mature miRNA, processed from the miRNA precursor by Dicer.	name, accession, evidence, experiment, similarity, sequence	**Precursor, SeedFamily**

Species	The taxonomic species having miRNAs encoded in the genome.	abbreviation, name, division, taxonomy, genome_assembly	**Precursor**

PrecursorFamily	The miRBase grouping of precursors into families.	name, accession, description	**Precursor**

GenomeContext	Other gene models overlapping the miRNA precursor in genome.	overlap_sense, overlap_type, transcript_source, transcript_name	Precursor

GenomePosition	The position of the miRNA precursor in the genome.	xsome, contig_start, contig_end, strand	Precursor

PrecursorCluster (*)	A grouping of precursors occurring close to each other in the genome - presumably transcribed together.	Name	**Precursor**

SeedFamily (*)	A grouping of mature miRNAs based on the 6mer seed (bases 2-7) or 7mer seed (bases 2-8).	name, sequence	**Mature**

Paper	Papers related to the annotation and identification of a miRNA as reported in miRBase.	medline, title, author, journal	**Precursor**

**miRMaid data models**: miRMaid restructures data in miRBase to yield a set of core data models. The data for the two models highlighted with a (*) is not readily available from miRBase data but is automatically computed when miRMaid is deployed. Each model is semantically related and connected to other models as listed in the last column of the table. Relationships highlighted in bold denote a 'many' relationship: a species has 'many' miRNA precursors, while a miRNA precursor is only related to 'one' species. The attributes and relations on each data object can be accessed in an object-oriented manner (see Figure 5 for an example).

**Figure 2:**
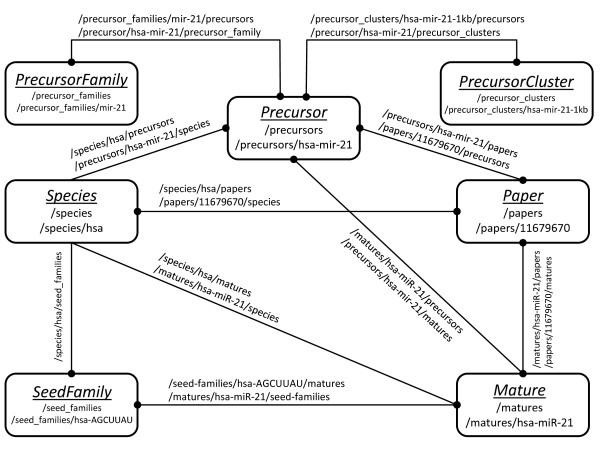
**Resource map**. Each data model (i.e. 'Precursor') in miRMaid has resource URLs for listing all objects (/precursors) or a single object (/precursors/hsa-mir-21). Relationships (denoted by edges in the figure) between models are captured by nested resource URLs (/matures/hsa-miR-21/papers). A solid circle at the end of an edge denotes a 'many' relationship. For example, a species 'has many' precursors (/species/hsa/precursors), while a precursor is related to only 'one' species (/precursors/hsa-mir-21/species).

All models are also exposed on the web as read-only RESTful resources, rendering HTML to researchers (using web browsers) and XML or FASTA representations to computer programs. Figure 2 illustrates the miRMaid resources, the associations between resources and how they are addressed by an URL. miRMaid (using RoR) ships with a lightweight, but efficient, web server that can be loaded from the command line, but miRMaid is also easily integrated with an existing Apache web server.

### Modular design

**Figure 3:**
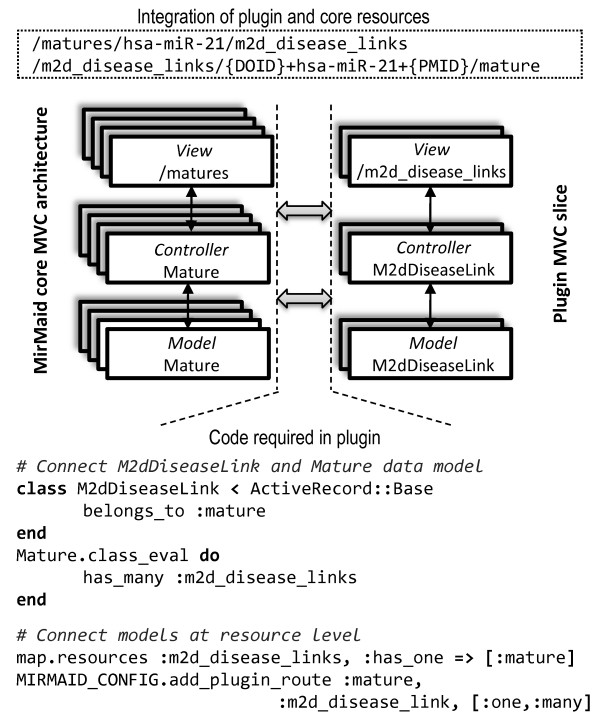
**Plugin integration**. A miRMaid plugin is implemented as an isolated MVC slice (an 'Engine' in the Ruby on Rails framework). The plugin defines its own data models and the relationships between these models. The integration (model and resource relationships) between the miRMaid core framework and the plugin is configured inside the plugin. The core framework provides hooks where a plugin can register itself. In the example above, the miR2Disease plugin defines two data models, M2dDisease and M2dDiseaseLink, where only the M2dDiseaseLink integrates directly with the core framework (a 'one-many' relationship with the Mature data model and resource). The effect of this integration is that M2dDiseaseLink objects are connected to Mature objects and that these relationships can be queried directly through the data models or by using RESTful resource URLs.

A central feature of miRMaid is its modularity. It has a structured, but simple application interface (RESTful web-service or the Ruby object-relational layer) and can be loosely coupled as an independent data component in existing systems. Furthermore, miRMaid is built as a framework that is easy to extend with new data and functionality. We have designed a plugin architecture, where the core miRMaid framework works independently of activated plugins. The plugins can dynamically integrate with and extend miRMaid data and functionality without making changes to the core application. It is a simple procedure to develop an extension or plugin to miRMaid that introduces new data models and resources integrated with the core miRMaid framework (Figure 3). The result is a modular web application, where the core miRMaid framework can be dynamically extended with plugins to provide a unified browsing experience and application interface. Please, refer to the result section for an example of how the plugin integration works in practice.

## Results and Discussion

### Maintenance and lifecycle of miRMaid

miRBase is the data source of the core miRMaid framework. With every data release of miRBase there will be a corresponding public version of the miRMaid web service) while older miRMaid versions will be kept available for a limited time period. Besides being a public web service, miRMaid can easily be installed locally. When a new version of MirBase is released, a local installation can be updated simply by reinstalling the miRMaid framework (together with optional plugins) using a single command on the command line. The source code for miRMaid is under the LGPL license and utilizes the Git multi-user versioning system (accessible via http://www.github.com). When changes are committed and released in the miRMaid project repository, it is a simple task to pull the changes and update a local miRMaid installation.

In miRMaid, there are unit tests for all models and RESTful resources. This is done to assist development and so that end-users can verify that their local miRMaid installation behaves as expected. The test suite can be run from the command-line. Plugins must also specify tests for models, RESTful resources and connections between the plugin and the core framework. The plugin unit tests are straightforward to implement and they are automatically evaluated together with the core test suite in miRMaid.

### RESTful clients

**Figure 4:**
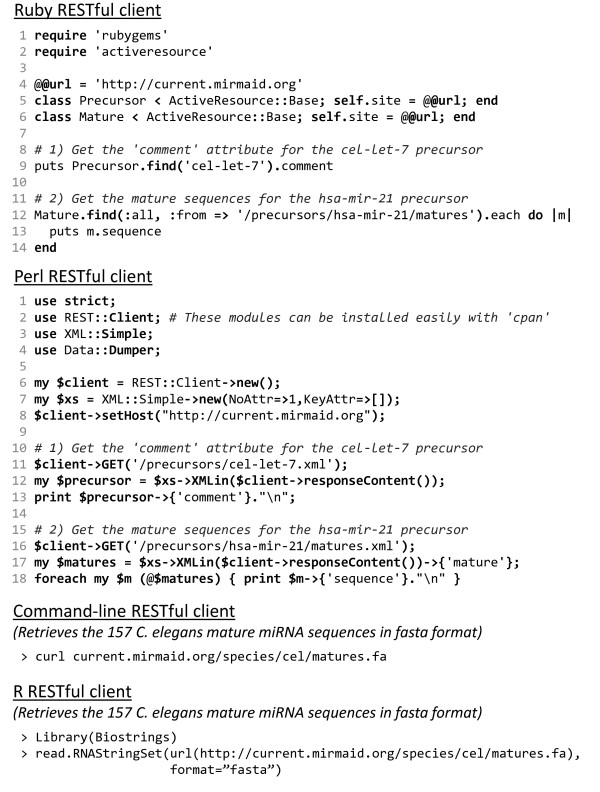
**RESTful clients**. RESTful clients can be implemented in most programming languages. Listed above are two examples in the Ruby and Perl programming languages. Both programs perform the same tasks: getting the 'comment' attribute for the cel-let-7 miRNA precursor and getting the mature miRNA sequences (hsa-miR-21 and hsa-miR-21*) for the hsa-mir-21 miRNA precursor. Both programs use standard libraries to issue HTTP GET requests and to parse the resulting XML documents. The final two examples demonstrate how miRMaid's FASTA sequence rendering capability can be used. We use the R statistical framework [21] and the 'curl' command-line program to issue a HTTP request to retrieve all *C. elegans *mature sequences in FASTA format.

A major benefit of a RESTful web service is the simplicity by which programs or other web services can retrieve information. Querying a RESTful web service only requires that the program is able to generate a HTTP request to the URL that specifies the resource and then parse the response document - most programming languages have such features readily available. miRMaid can generate HTML and XML response documents for all resource URLs and FASTA documents where it is appropriate. XML documents are suited for computer programs and they are easily handled and parsed in most programming languages. In Figure 4 we give two examples of RESTful clients implemented in the Ruby and Perl programming languages. Both programs perform two simple tasks: 1) retrieving the comment attribute for the cel-let-7 precursor, and 2) retrieving the sequences for the two mature miRNAs (hsa-miR-21 and hsa-miR-21*) in the hsa-mir-21 precursor. In Figure 4, we have also included two examples to illustrate the simplicity of the RESTful interface. We use the R statistical framework [21] and the 'curl' command-line program to issue a HTTP request to retrieve all *C. elegans *mature sequences in FASTA format. Furthermore, a normal web-browser can be used as a RESTful client to inspect the XML and FASTA response documents for a given URL. There is currently no widely adopted web service description standard for RESTful services. Until a standard has been adopted, the resource API for a given miRMaid instance (including installed plugins) is dynamically documented via the URL http://current.miRMaid.org/described_routes.txt (also available as an XML document). This feature is further documented on the miRMaid community site.

### Local Ruby clients with direct access to data models

**Figure 5:**
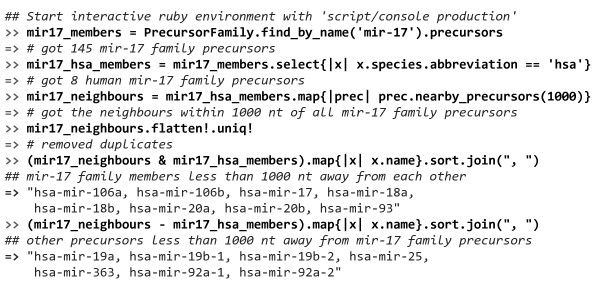
**Ruby data models**. In a local miRMaid installation, the data models can be queried directly without the overhead of the HTTP protocol and network communication. The figure lists an interactive Ruby IRB session where the data models are queried to analyze the genomic clustering of human mir-17 family members.

The second leg of miRMaid is the object oriented model layer. With a local miRMaid installation data can be accessed efficiently through a Ruby program without the overhead of HTTP protocol and network communication that is associated with the REST interface. miRMaid uses the RoR object-relational mapping library called ActiveRecord. This library provides an intuitive way to find objects, retrieve attributes and to navigate between associated models. In Figure 5, we provide an example of how the models can be queried interactively in a Ruby IRB session. We start out by retrieving all 8 human precursors in the mir-17 precursor family. Next, we identify all precursors in a neighborhood of +/- 1000 nucleotides. These nearby precursors are finally grouped into mir-17 family members and non mir-17 family members. This is a very simple example yet it illustrates how the data models can be queried swiftly in an intuitive manner.

### miRMaid plugins

As detailed earlier, data and functionality in miRMaid can be extended by plugins. We have developed a proof-of-concept plugin using data from the miR2Disease web service [22]. The plugin extends miRMaid with two data models and RESTful resources: diseases and disease links. A disease link associates a mature miRNA and a disease and it carries information about the association, for example PubMed reference and target genes. A specific disease instance can be reached using the URL,/m2d_diseases/DOID, where DOID is the Disease Ontology identifier. Disease links are identified by a concatenation of DOID, mature miRNA name and PubMed ID. Figure 3 demonstrates how the plugin connects with miRMaid to integrate the disease link model and resource with the miRMaid mature model and resource. The plugin should also define HTML representations for the resources that are being introduced. These plugin HTML representations are accessible from a web browser and are automatically integrated in the menu layout of the miRMaid web site. The net effect is a complete integration of miRMaid and plugin in both the web site and application interface. We host a public version of miRMaid with example plugins activated at http://plugins.mirmaid.org.

## Conclusion

First of all, miRMaid is a software framework aiming at easing the manual workload for researchers when doing computational analyses involving miRNA data. miRMaid provides a uniform, intuitive and flexible application interface that is independent of programming language. miRMaid is designed to live as a public service as well as being installed locally. The public service should be used when doing a simple and quick analysis and for integration with other web services. The local installation (using the Ruby data models) is recommended when a more data extensive analysis is needed. miRMaid is open-source software and users can contribute to the framework through the public source code repository or they can develop a miRMaid plugin that can be shared with the rest of the community. Furthermore, individual users or labs can integrate private data as miRMaid plugins or they can couple existing information systems loosely to miRMaid using the RESTful API.

We believe that the miRMaid platform can pave a new and exciting way for scientists to share data and programs that involve miRNAs. miRMaid follows a design philosophy that web services and resources should be able to integrate: web services should participate in the web instead of merely living on the top of it. We envision that if new data resources are released as miRMaid plugins, or at least follow the RESTful design principles for web services, then this would be a big step towards a global integration of miRNA data. By developing miRMaid we hope that such an effort can be coordinated not only by huge centralized software development teams at Ensembl and the UCSC genome browser, but also by a community that shares a common scientific interest.

## Availability and requirements

• Project name: miRMaid

• Project home page: http://www.mirmaid.org.

• Operating systems: Server software: Linux and Mac OSX, Client software: Platform independent.

• Programming language; Server software: Ruby. RESTful clients: most modern programming languages.

• Other requirements; Database management system: PostgreSQL, MySQL or SQLite. Other minor requirements are detailed at http://www.mirmaid.org.

• License: Free for academic and commercial users under the GNU Lesser General Public License (LGPL).

• Public servers: A public server running the current miRMaid release can be found at http://current.mirmaid.org and a server instance with example plugins activated can be found at http://plugins.mirmaid.org.

## Competing interests

ML and SK are employees of Santaris Pharma A/S, a biopharmaceutical company developing RNA-based medicines.

## Authors' contributions

AJ designed and implemented most of the software and drafted the manuscript. ML conceived of the project, designed and tested the software and helped draft the manuscript. All authors read, helped draft and approved the final manuscript.
